# To save or not to save: Knowledge, attitude, skills and effects of an experimental intervention on advancing first aid skills in high school students in Hue City, Vietnam

**DOI:** 10.1371/journal.pone.0322505

**Published:** 2025-04-29

**Authors:** Le Duc Huy, Pham Thanh Tung, Dinh Thanh Tra, Le Nguyen Quynh Nhu, Nguyen Tuan Linh, Tran Xuan Tien, Nguyen Vu Phuong Thao, Trinh Thi Le Vy, Tran Thi Hang, Huu Hai Hoang, Vo Van Khoa, Nguyen Thi Anh Phuong, Bui Phuong Linh

**Affiliations:** 1 College of Health Sciences, VinUniversity, Hanoi, Vietnam; 2 Department of Epidemiology, Harvard T. H. Chan School of Public Health, Boston, Massachusetts, United States of America; 3 Research Advancement Consortium in Health, Hanoi, Vietnam; 4 Department of Orthopedics, Cho Ray Hospital, Ho Chi Minh, Vietnam; 5 Department of Infectious Disease Prevention and Control, Dak Lak Provincial Center for Disease Control, Dak Lak, Vietnam; 6 Tianjin University of Chinese Medicine, Jinghai District, Tianjin, P. R. China; 7 Department of Radiology, University Medical Shing Mark Hospital, Dong Nai, Vietnam; 8 Department of Medical Genetics, University of Medicine and Pharmacy, Hue University, Hue, Vietnam; 9 Tam Anh Ho Chi Minh City General Hospital, Ho Chi Minh, Vietnam; 10 Faculty of Nursing, Hue University of Medicine and Pharmacy, Hue University, Hue, Vietnam; 11 Faculty of Public Health, Danang University of Medical Technology and Pharmacy, Danang, Vietnam; 12 Department of Obstetrics and Gynecology, University of Medicine and Pharmacy, Hue University, Hue, Vietnam; 13 Faculty of International Education, University of Medicine and Pharmacy, Hue University, Hue, Vietnam; 14 Office of Science-Technology and International Relations, University of Medicine and Pharmacy, Hue University, Hue, Vietnam; Center for Research and Technology Transfer, VIETNAM

## Abstract

Unintentional injury is the leading cause of death in adolescents especially in low-middle-income countries including Vietnam. However, there is limited evidence on first aid (FA) knowledge, attitude, and skills among Vietnamese adolescents. Our study aims to assess the knowledge, attitudes, and skills regarding FA; and evaluate how these domains improve after implementing FA training programs among high school students in Hue city. A single-group pre-post-intervention study was conducted on high school students between June and September 2020 in Hue City, Vietnam. The training materials were developed based on international guidance on FA and adjusted to a low-middle-income country context, particularly in Vietnam. The training program provided students with essential knowledge and skills focused on the management of common injuries in Vietnam to enhance a positive attitude toward FA. The students’ responses on FA knowledge and attitude were collected using self-reported questionnaires and FA skills were evaluated by skill checklists. At baseline (n = 806), the average score of FA knowledge, attitude, and skills over a 100-point scale were 44.0 (95% CI: 38.6, 49.5), 59.4 (95% CI: 55.5, 63.4) and 39.2 (95% CI: 35.8, 42.6) respectively. Following the intervention phase (n = 106), which included a 3-hour FA training session, a significant increase in FA skill scores was observed, with a mean difference of 37.0 points (SE: 1.6). The findings demonstrated the feasibility and significant impact of a short course of FA training on improving FA skills among high school students. The results of the multivariable regression model showed baseline scores of FA attitude, FA knowledge or having previous FA training were not significantly associated with the improvement in FA skill scores in this pre-post intervention. The study findings highlighted the low level of FA knowledge, attitude, and skills; and provided evidence of the effectiveness of FA training in improving FA skills among high school students in Hue City, Vietnam.

## Introduction

Unintentional injuries are the top cause of death in children and young people, leading to a global public health issue. It is estimated that over 1600 children younger than 19 years die every day from these injuries worldwide [1]. In Vietnam, the national report of the school-based student health survey showed that more than 20% of students experienced severe injuries in 2019 [2]. The injured children and their families usually experience either extreme emotional or financial costs [1].

First aid (FA) is widely recognized as an effective approach to improving survival rate, minimizing injury consequences, reducing pain, and making victims more comfortable in resuscitative or non-resuscitative situations [3,4]. FA refers to a sequence of initial care offered for an acute or illness [5]. The activities are performed by anyone in any situation to achieve goals of preserving life, alleviating suffering, preventing further illness or injury, and promoting recovery [5]. Previous studies showed that rapid response with FA can improve the rates of survival to discharge, 1-month survival, and 1-year survival among out-of-hospital cardiac arrest [6]. Therefore, the widespread knowledge of FA skills at work and schools is essential to reduce the burden caused by unintentional injury [3].

American Heart Association (AHA) and World Health Organization (WHO) recommended the implementation of FA training, including CPR training in early childhood since children have sufficient ability to conduct some basic FA such as the call for support [7]. The importance of FA training in adolescents has been emphasized for several reasons: Students play such an important role as a bystander on or off campus. Thus, children with adequate first-aid skills can provide effective medical support for not only injured children but also for patients suffering cardiac arrests. Several studies indicated that students are highly willing to perform FA, which increases the accessibility of FA in community [8,9]. Children can be active health educators who might introduce knowledge or principles regarding FA to their families and the public.

Although first-aid training for children was well recognized and emphasized, there was no consensus on the optimal content and duration of first-aid training. A previous study highlighted that simplified content plays an important role in teaching FA to children [10]. Han et al. suggested education goals of FA courses for children and students should focus on basic skills such as calling numbers, recovering position, wound and burn care and stopping bleeding, managing wounds, and burn care [11]. In addition, with the limited time available in the school, the duration of the training course needs to be calculated carefully to facilitate the student’s participation [11].

In Vietnam, FA training is often integrated with other courses in the high school curriculum and mainly focuses on resuscitation skills with limited practice sessions. Several previous studies in Vietnam indicated poor knowledge and practice regarding FA in undergraduate students [12,13]. However, no studies evaluated the knowledge and practice of FA and assessed the effects of FA training on adolescents in Vietnam. To bridge the gap, we carried out the studies with the following aims: (1) To assess the knowledge, attitudes, and skills regarding FA among high school students; (2) To evaluate the effectiveness of our experimental training program to improve the FA skill among high school students.

## Materials and methods

### Study design

We conducted a single group before-and-after intervention study on high school students in Hue City, Vietnam. Our study included two phases: (1) the baseline phase focusing on assessing the knowledge, attitude, and skills regarding FA conducted from June 1 2020 to July 1 2020, and (2) the intervention phase providing the FA training program during August 17 and September 18, 2020.

### Study sites

**Fig 1:**
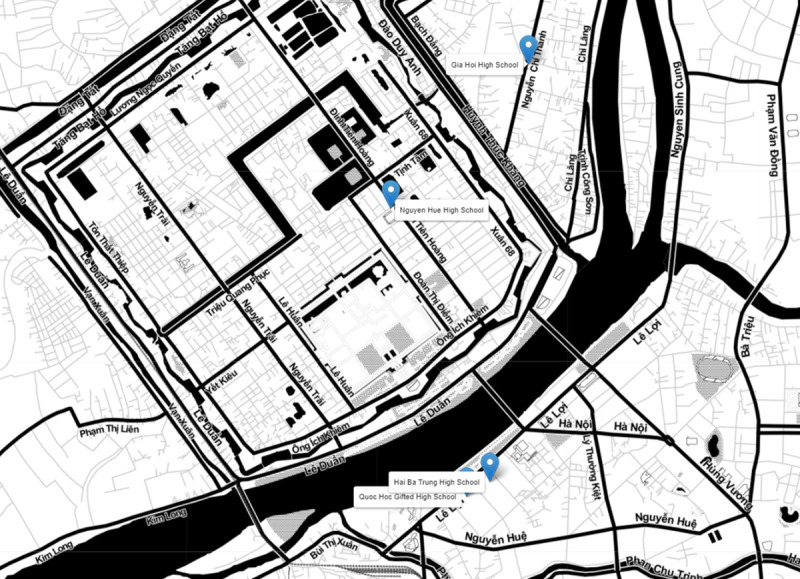
The location of four selected schools (Base map and data from OpenStreetMap and OpenStreetMap Foundation. The map contains information from OpenStreetMap and OpenStreetMap Foundation, which is made available under the Open Database License).

This study was carried out in Hue City, located in Central Vietnam. Hue City is a medium-sized city and a major education and healthcare center in Vietnam. In comparison with the big cities with higher living standards and huge investments in school facilities, such as Ha Noi City, Ho Chi Minh City, and Da Nang City, Hue City shares similar characteristics with the majority of cities in Vietnam. Therefore, the development of training programs in Hue City would facilitate future applications to other cities in Vietnam. Additionally, the University of Medicine and Pharmacy, Hue University is one of the three most long-lasting medical institutions in Vietnam, which have strong collaborations with high schools in Hue city. The logical and technical support from experts in Hue City plays an important role in developing, implementing, and evaluating the training programming in the study. Therefore, Hue City served as an appropriate place to implement the study on FA training (Fig 1). In 2019, over 8000 students grade 10 and 11 studied in 11 high schools (4 in the North and 7 in the South) in Hue city [14].

### Study participants and sampling

**Fig 2:**
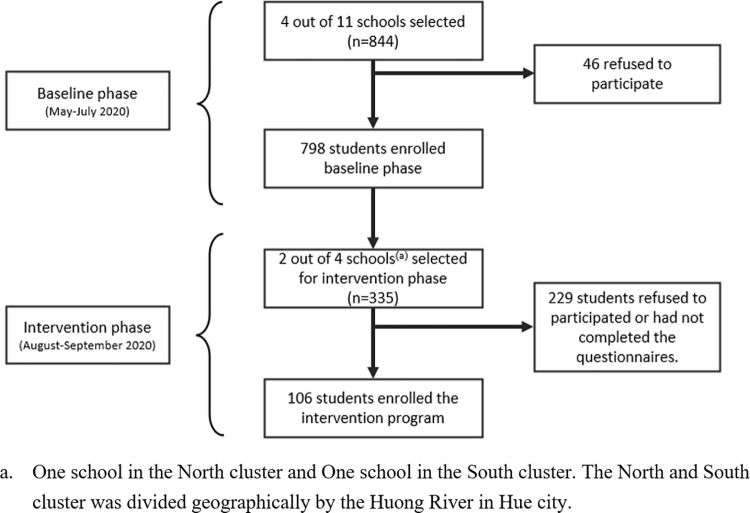
Flow chart of study participation.

We included high school students in grades 10 and 11 in the academic year 2019–2020 in our study sample. Fig 2 showed the flow of participant selection and recruitment.

At baseline, we randomly selected 4 out of 11 high schools in Hue city (Fig 1). The Perfume River (Huong River) separates the city into 2 regions: the traditional town in the Northern river and the more modern city in the Southern river [15]. Due to the significant difference in socioeconomic status between the 2 regions, we randomly selected 2 schools in the North and 2 schools in the South (Fig 1). In each selected school, we randomly selected 8–10 students in each class of grade 10 or 11 to reduce potential selection bias. Because of the school policy on protecting student information, we cannot access the full list of information of students to generate a random list of participants. Hence, we provided teachers with detailed instructions on random selection in research training and asked them to send us the list of selected students in each class. The final calculated sample size was 844. The sample size calculation, inclusion criteria, and sampling methods of the baseline phase were described in detail elsewhere [8]. Since 46 students refused to be involved in the study, we included 798 participants in the baseline phase.

For the intervention phase, we randomly selected two schools (one from the North and one from the South of Huong River) from the four selected schools at baseline. We sent out invitations to 335 students in the two selected schools; however, 229 students did not enroll in the phase or complete the assessment. Most of these dropouts reported reasons related to the COVID-19 outbreak. Since high school students have different schedules across classes and grades, we provided multiple training sessions to accommodate their participation. Unfortunately, the COVID-19 outbreak in Hue City led to the implementation of social distancing measures, including school closure. Thus, the final sample size for analysis of the intervention phase was 106 students.

### Measures

#### First aid knowledge, attitude, and skills.

**The questionnaires on FA knowledge and attitude**

We developed a questionnaire focusing on the knowledge and attitude of FA procedures for common injuries in Vietnamese adolescents according to the data on the national school health survey [16]. With this focus, an expert panel including 3 senior lecturers on nursing and 1 specialist in intensive care was established to review the literature and create the first draft of the questionnaire. Regarding the readability of questions, we conducted a group discussion with 4 high school students (2 grade-10 students and 2 grade-11 students) to revise the words, sentence structure, and change the order of questions.

The final questionnaire on FA knowledge includes 35 items that assess primary assessment, ambulance calling, CPR, bleeding control, joint injury management, fracture mobilization, and burn management. With a score ranging from 0.5 to 1.5 points for every correct answer, the total score of the FA knowledge section was 34.5 points. For FA attitude, we developed 12 five-point Likert-scale items ranging from 1 (totally disagreed) to 5 (totally agreed). The total score of FA attitude is 48 points. To facilitate the comparison among the scores of knowledge, attitude, and skill, we standardized them on a scale ranging from 0–100 points. All answers of students on FA knowledge and attitude were recorded via the smartphones or tablets at baseline. Table S4 shows the detailed score of the FA knowledge and attitude.

### The checklist of FA skill

The checklist of FA skills was designed according to key topics in the FA knowledge questionnaire (S7 Table). To create the checklist, the expert panel discussed and reviewed previous studies and FA guidelines of the Vietnamese Ministry of Health, Vietnamese Red Cross [17,18] and other well-known international organizations [19,20]. The final checklist included 24 items focusing on primary assessment and ambulance call (9 items), chest compression (5 items), ventilation (6 items), and stopping bleeding (4 items). Every item was rated at 3 levels: poor performance (0 point), good performance (1 point), and excellent performance (2 points). The scores of FA skills ranged from 0 to 48 points (S2 Table). The checklist was evaluated by the 4th and 5th-year medical students at the baseline phase and immediately after the training phase. The detailed score of the FA skills in the checklist was presented in S6 Table.

#### Demographic and background factors.

In addition to FA knowledge, attitude, and skills, we asked participants about demographic information such as gender, grade, experience of prior FA training, history of injuries, and the sources of information on FA learning.

### Development of first aid training program as the intervention tool

We developed a FA training program to improve student ability regarding injury management and safety during implementing FA. First, we established the technical advisory committee (3 senior nurses and 1 Critical care specialist) to develop training materials on FA. Next, the committee organized 2 meetings with 90 minutes to review materials based on the scientific guidelines (e.g., IFRC, UK cross red, US cross red, and other Vietnamese Red Cross and Vietnamese Ministry of Health) and defined the priority training topics. All English training materials were translated forward and backward for validation. The draft training protocol was developed based on the consensus within the technical committee. Then, the technical committee revised and finalized the training protocol based on the feedback from students, collaborators, and project teams in a pilot training for 20 newly graduated high school students.

The details of the FA training protocol were described in S1 Appendix file. In brief, a 180-minute in-class lesson was designed to improve the knowledge, attitude, and skills of FA of 15- to 17-year-old students. The lessons were delivered by 2 senior lecturers in Nursing with assistance from senior medical students.

The training lessons were divided into theoretical and practical sessions:

- The theory session included (1) Warming up: emergency situations, root analysis of the risk and prevention measures (15 minutes); (2) The importance of readiness for FA (10 minutes); (3) FA training skills based on 4 accident scenarios (60 minutes); (4) Wrap up (15 minutes). The learning materials, of course, included a FA manual designed for high school students, a PowerPoint presentation, and laptops that allowed the students to watch videos on how to implement circulation resuscitation and interact with the instructor via a FA knowledge quiz.
- In the practical session, the lecturer and teaching assistants demonstrated the steps to perform the primary assessments, chest compression, ventilation, and stopping bleeding. The group-based learning (4–5 students per group) was adopted to facilitate students in practicing skills with training equipment and receiving feedback from instructors and their peers. The length of the practical session was 90 minutes. We used the manikins and bandages to demonstrate and support students in practicing FA skills.

### Study procedure and data collection

#### Baseline phase.

We worked closely with the schools to obtain consent from the parents and students who were invited to the study. The participants in the baseline were explained about an evaluation procedure. During the first section, a self-reported electronic questionnaire on FA knowledge and attitude was given to the students to complete in the classroom. Under the guidance of the research assistants, participants used laptops, tablets, or cellphones to access the questionnaire. In the second section, participants underwent 4 stations to evaluate their FA skill of primary assessment, chest compression, ventilation, and stopping bleeding. After finishing the whole assessment, the student received a water bottle as compensation for participating in the study. The estimated time for the two assessment sections was around 40–45 minutes.

#### Intervention phase.

The study participants who agreed with parenteral permission, were informed of the location and time of training classes via either the telephone or email. We carried out 4 training classes from August 17 2020 to September 18 2020. The training class lasted for 180 minutes and included theory combined with skills.

The classes were led by two senior lecturers in nursing who have extensive experience in first-aid training at the University of Medicine and Pharmacy, Hue University.

To ensure the quality of the training course, the class was limited to 25 students and divided into five groups (4–5 students per group). Each study group was supported by facilitators who were 4^th^- or 5th-year medical students and thoroughly trained with a standardized protocol. After completing the practical sections, the students were assessed for FA skills immediately.

This study followed the EQUATOR guideline for cross-sectional studies (S2 Appendix).

### Data analysis

We used the paired t-test or Wilcoxon if appropriate to evaluate the effects of training on the FA skills among students. To identify the factors associated with differences in skills scores after training, multivariable regression analyses were performed (S3 Appendix). The primary outcome of the model was the changes in FA skill scores between before and after training. The independent variables included gender, grade, the experience of prior FA training, history of injuries, and sources of information on FA learning.

Besides, we performed the exploratory factor analysis (EFA) to evaluate construct validity, which identified the possible latent variables and uncovered the structure of items in a FA attitude. To assess the statistical power of our study design in detecting the difference in FA skill scores before and after the intervention, we conducted a post hoc power analysis using the pwr.t.test in R. Given a score difference of 36.98, a standard deviation of 16.21, and a sample size of 106 pairs, the result showed that our study’s statistical power is 100%. This confirmed that our analysis was well-powered to detect a significant difference in FA skill scores before versus after intervention [21]. The data were analyzed by STATA MP (version 16.0) and visualized by R programs (version 4.2.2).

### Ethical considerations

The study protocol was approved by the Institutional Review Board (IRB) of Hue University of Medicine and Pharmacy (No. IRB: H2020/057). We also obtained approval to implement the study from the executive boards of the four surveyed high schools. All eligible students and their parents were informed by the research team about the study context, objectives, and procedure, along with the minimal risks associated with enrollment and the confidentiality of their data before entering the study. Students who agreed to enroll in the study submitted written informed consent to designated research staff. The process of obtaining consent was witnessed by staff members and high school teachers. All consent forms were documented at the Student Union Office of Hue University of Medicine and Pharmacy. The students’ parents were contacted to inform them about the study information and obtain their consent for the students’ participation verbally via phone call. All participation was voluntary, and participants could withdraw from the study at any time.

## Results

Table 1 presents the characteristics of study participants during the baseline phase. At the baseline of the study, the majority of students were male (63%). The proportions of grade 10 students (54.4%) and grade 11 students (45.6%) were approximately equal. The prevalence of students receiving the previous FA training was less than 13%. The majority of students received FA information from the internet (82.8%) and teachers (63.8%).

**Table 1:** Demographic of study participants during baseline phase.

Characteristics	n (%)
**High schools**	
Nguyen Hue high school	148 (18.5)
Quoc Hoc gifted high school	187 (23.4)
Hai Ba Trung high school	232 (29.1)
Gia Hoi high school	231 (28.9)
**Gender**	
Male	505 (63.3)
Female	293 (36.7)
**Grade**	
10	434 (54.4)
11	364 (45.6)
**Prior FA training**	
No	717 (89.8)
Yes	81 (10.2)
≤ 1 year training	29 (35.8)
>1 year training	34 (42)
Don’t know	18 (22.2)
**Sources of FA information if received (Multiple answers)**	
Internet	661 (82.8)
Relatives	375 (47)
Friends	272 (34.1)
Television	420 (52.6)
Teachers	509 (63.8)
Others (movies, books…)	30 (3.8)
**Injury experience in the past 12 months**	
No	665 (83.3)
Yes	133 (16.7)

Scores of knowledge, attitude, and skills were rescaled on the range from 0 to 100 points.

Table 2 presents the results of iterative principal factor analysis with an oblique Promax rotation after the items related to FA attitudes with the highest loading factor <0.4, or uniqueness >0.5 were removed. The analysis showed that items related to FA attitudes were classified into three subgroups, including confidence in performing FA (3 items), demands of FA training (2 items), willingness to perform FA (2 items) (S1 Fig, S1 Table).

**Table 2:** Factor loading of items in the attitude towards FA scale at baseline.

Items	Factor1	Factor2	Factor3	Uniqueness
Confidence to perform the FA for people who need help.	0.82			0.31
Confidence to perform all types of FA skills.	0.80			0.38
Confidence to perform some FA skills.	0.76			0.41
Learning FA is very important.		0.83		0.28
FA training for a high school student is necessary.		0.74		0.44
If the victim is a stranger, I will provide the FA.			0.75	0.43
I will perform the FA when other people are present.			0.65	0.48

Table 3 shows that the average baseline scores of FA knowledge, attitude, and skill over a 100-point scale were 44 (95%CI: 38.6, 49.5), 59.4 (95%CI: 55.5, 63.4), and 39.2 (95%CI: 35.8–42.6), respectively. The overall scores in FA knowledge, FA attitude, and FA skills were similar between male and female students. Regarding the score of specific subdomains in FA knowledge and skills, we found females had statistically higher scores than males in the knowledge of joint injury management (40.3 (95%CI: 32.6, 48.0) vs 33.5 (95%CI: 27.2, 39.7), p < 0.001) and the skill of chest compression (22.7 (95%CI: 1.7, 47.1) vs 15 (95%CI: 2.5, 27.5), p < 0.001). In contrast, males showed a higher score in the knowledge of burn management than females (40 (95%CI: 32.3, 47.6) vs 42.6 (95%CI: 40.0, 45.2), p = 0.012). The other scores of subdomains in FA knowledge (primary assessment, cardiopulmonary resuscitation (CPR), bleeding control, fracture mobilization) showed no statistical difference. Similarly, the score of FA attitude and subdomains of skills (Primary assessment, Ventilation circulation, Bleeding control) were observed with no statistical difference.

**Table 3:** Knowledge, attitude, and skills of participants at the baseline phase (N = 798).

	Total Mean (95%CI)	Female Mean (95%CI)	Male Mean (95%CI)	p-value
**Knowledge**	44 (38.6, 49.5)	44.1 (39.2, 49.1)	43.9 (38.2, 49.7)	0.528 ^b^
Primary assessment and call ambulance	44.8 (43, 46.7)	44.3 (41.6, 47.1)	45.2 (43.1, 47.3)	0.328 ^b^
Cardiopulmonary resuscitation (CPR)	44.7 (38.4, 50.9)	44 (40.8, 47.2)	45.1 (36.3, 53.9)	0.571 ^b^
Bleeding control	43 (32.7, 53.3)	42.9 (33.2, 52.7)	43 (31.8, 54.2)	0.894 ^b^
Joint injury management	36.1 (28.9, 43.4)	40.3 (32.6, 48.0)	33.5 (27.2, 39.7)	**<0.001** ^c^
Fracture mobilization	49.4 (39.8, 59)	52.4 (41.2, 63.6)	47.5 (39.4, 55.6)	0.11 ^c^
Burn management	41.6 (37.2, 45.9)	40 (32.3, 47.6)	42.6 (40.0, 45.2)	**0.012** ^c^
**Attitude** ^a^	59.4 (55.5, 63.4)	59.2 (54.7, 63.7)	60 (56.7, 63.3)	0.116 ^b^
**Skill**	39.2 (35.8, 42.6)	40.3 (33.7, 46.9)	38.5 (36.7, 40.2)	0.295 ^b^
Primary assessment	49.3 (44.5, 54)	49.8 (46.2, 53.3)	49 (43.0, 55.0)	0.524 ^b^
Chest compression	18 (0.9, 35.2)	22.7 (1.7, 47.1)	15 (2.5, 27.5)	**<0.001** ^c^
Ventilation circulation	24.4 (20.3, 28.4)	25.1 (17.4, 32.8)	23.9 (13.6, 34.1)	0.372 ^c^
Bleeding control	62.7 (53.6, 71.9)	61.7 (53.6, 69.7)	63.4 (53.0, 73.8)	0.296 ^c^

The scoring of knowledge, attitude, and skills were standardized on a scale ranging from 0 to 100 points (S2 Table).

^a^Number of attitude-related items were exhausted based on the result of EFA (The selected items of FA attitude are presented in S5 Table)

^b^Independent T test

^c^Mann-Whitney-Wilcoxon test

Table 4 describes the characteristics of study participants during the baseline phase. In the intervention phase, the proportion of female students (62.3%) was dominant, and the percentage of grade 10 students participating in the intervention program (76.4%) increased remarkably compared to the baseline period. Other characteristics of participants in the intervention phase were similar to the baseline phase.

**Table 4:** Demographic of study participants during the intervention phase (N = 106).

Characteristics	n (%)
**High schools**	
Nguyen Hue high school	53 (50)
Quoc Hoc gifted high school	53 (50)
Hai Ba Trung high school	NA
Gia Hoi high school	NA
**Gender**	
Male	40 (37.7)
Female	66 (62.3)
**Grade**	
10	81 (76.4)
11	25 (23.6)
**Prior FA training**	
No	93 (87.7)
Yes	13 (12.3)
≤ 1 year training	5 (38.5)
>1 year training	6 (46.2)
Don’t know	2 (15.4)
**Sources of FA information if received (Multiple answers)**	
Internet	93 (87.7)
Relatives	56 (52.8)
Friends	36 (34)
Television	64 (60.4)
Teachers	74 (69.8)
Others (movies, books…)	5 (4.7)
**Injury experience in the past 12 months**	
No	92 (86.8)
Yes	14 (13.2)

Scores of knowledge, attitude, and skills were rescaled on the range from 0 to 100 points.

**Fig 3:**
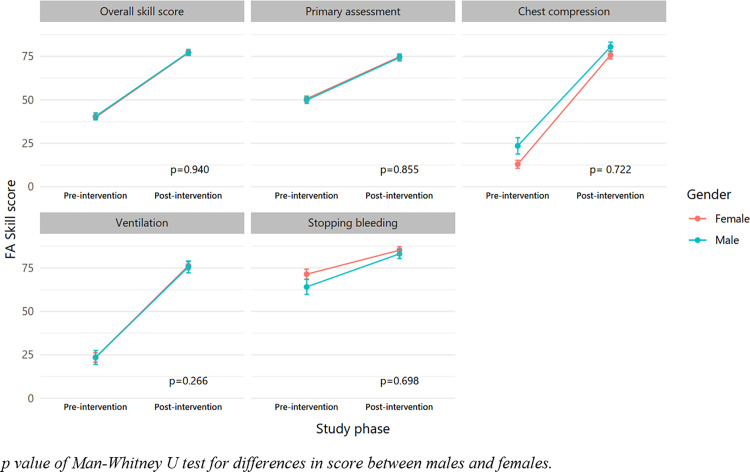
The score of FA skill at baseline and after intervention among male and female participants (n = 106).

Fig 3 presents a considerable increase in FA skill scores before and after the intervention. We observed a similar increase in overall FA skill score, primary assessment, and ventilation among subgroups of sex. Compared to female students, male students showed a greater improvement in FA skills score for Chest compression (48.6 in males vs 52.5 in females), but a smaller improvement in FA skills score regarding stopping bleeding (8.9 in males vs 5.0 in females) (S3 and S4 Tables). Similarly, there was no statistical difference in the improvement of FA skill scores between grade 10 and grade 11 students (S2 Fig).

Table 5 indicates the results of the multivariable linear regression model of factors associated with the difference in overall skill score between before and after FA training. The linear regression model found the improvement in FA skill score was negatively associated with being a grade-11 student (β: -3.7, 95%CI: -11.6 to 4.2) and having prior FA training (β: -6.1, 95%CI: -16.2 to 4). Conversely, male students showed a positive increase in FA scores compared to female students (β: 0.6, 95% CI: -6.1 to 7.3). However, our results showed that the schools, gender, grade, experience with FA training, FA attitude, and FA knowledge were not significantly associated with the increase in FA skill scores between pre- and post-intervention.

**Table 5:** Multivariable regression model of factors associated with the difference in overall skill score between pre- and post-intervention. (n = 106).

Characteristics	Change of skill score (post vs pre)	Coefficient	95%CI	p value
High schools				
Nguyen Hue high school	36.1 (17.8)	Ref	–	–
Quoc Hoc gifted High school	37.9 (14.6)	2.2	(-4.6, 9.1)	0.518
Gender				
Female	37.1 (14.5)	Ref	–	–
Male	36.7 (18.9)	0.6	(-6.1, 7.3)	0.862
Grade				
Grade 10	38.0 (15.5)	Ref	–	–
Grade 11	33.8 (18.4)	-3.7	(-11.6, 4.2)	0.358
Prior to FA training				
Yes	32.2 (21.7)	Ref	–	–
No	37.7 (15.3)	-6.1	(-16.2, 4)	0.233
Attitude score at baseline	NA	0.0	(-0.2, 0.2)	0.98
Knowledge score at baseline	NA	-0.2	(-0.5, 0.1)	0.299

*(Abbreviation: NA, Not applicable; Ref, reference)*

## Discussion

To our knowledge, the study provided the first evidence of the level of FA knowledge, attitude, and skills among high school students in Vietnam. Our results revealed the unsatisfactory levels of knowledge and skills regarding FA among surveyed students. Additionally, we evaluated the effects of a school-based FA intervention on narrowing the gaps in FA skills. The results of the intervention program provided promising opportunities to improve FA knowledge, attitude, and skills in Vietnamese high school students.

Our analysis showed the average score of FA knowledge was relatively low, with no subdomain reaching over 50 points on a standardized 100-point scale. This was consistent with previous studies in Egypt and Malaysia [3,22], which also reported poor knowledge scores or a high percentage of incorrect answers. In contrast, a survey in Norway showed a high level of knowledge regarding managing unresponsive adults in the secondary student population [23]. However, the comparison of results related to the FA evaluation still faced many challenges as there is a lack of standardized assessment tools to measure the FA knowledge and the variation of topics included in FA measurement between studies. Hence, there is an essential need to develop standardized and valid instruments that support the comparison of FA knowledge assessment across countries and globally. On the other hand, the students showed a low level of attitude towards FA, which posed barriers in improving the long-term FA knowledge and performing FA in practice [24]. Recently, the Vietnamese Ministry of Education and Training issued the first regulation on FA training for school teachers to disseminate FA knowledge within the school environment [966/QĐ-BGDĐT] [25]. However, the material was heavily focused on theory and provided limited guidance on improving the attitude of FA, which was a key factor in the accessibility of FA support in the community [24]. Previous experimental studies in Thailand and Malaysia highlighted the role of CPR training in promoting a positive attitude towards CPR among school children [26,27]. Therefore, our FA training program was developed to improve not only knowledge but also the attitude of students towards FA.

Among the FA skills assessed, chest compression and ventilation were recorded as the poorest skills (Table 3), whereas these skills are crucial for providing basic life support (BLS) in the community. Interestingly, these two skills witnessed the largest improvement after intervention (Fig 3). Our finding highlighted that with the appropriate short training, the students could rapidly improve their ability to perform the BLS. Previous evidence in Norway and Australia showed that the FA training course with the length ranging from 4 to 25 hours could significantly contribute to improving knowledge and retaining knowledge for up to 12 months [24]. Other factors might be associated with the performance of FA skills. Mathew et al carried out a study on 812 Indian school children and indicated that the student’s physical characteristics (height, weight, and BMI) were positively associated with the quality of performing CPR [28].

Our multivariable regression model showed that there was no significant association between the examined factors and differences in FA skills, including prior training, a widely recognized factor related to better FA knowledge and skill score. Our study found that the proportion of participants with the experience of FA training in pre and post-intervention phases accounted for 10.2% and 12.3%, respectively. These figures were slightly lower than the previous studies in Arab Saudi with 13.6% and Hong Kong with 14% [11,29] but higher than the result of a survey in China with 3% [9].

In the present study, the group of students with prior FA training did not score significantly higher compared to those without training after receiving the intervention. A previous study carried out by Kanstad et al. showed that students with previous BLS training in school had significantly higher confidence in BLS knowledge compared to those without this background [23]. A study in Vienna conducted on bystanders concluded that there was a clear relationship between the level of first-aid training and the quality of first-aid measures [30]. The quality of training could be an issue since current FA training in the Vietnamese high school syllabus mainly focuses on the theory and limitations of training equipment. A study in Hong Kong found that despite the higher score in groups with FA training, both groups showed a low knowledge score regarding the FA [31]. The small sample size in the intervention phase of our study may limit the statistical power of our analysis in detecting significant differences. Additionally, improving the accessibility of high-quality courses is crucial. In our study, we found that the source of information for FA training was mainly from the Internet (87.7%) and teachers (69.8%). This highlighted the crucial role of trusted sources, including social media and the internet in disseminating FA knowledge among students. Regular training for teachers and students played an important role in ensuring the quality of FA training and enhancing the long-term knowledge of FA. A recent study carried out by Cuijpers et al showed that a combination of learning the e-course combined with the intervention might provide a more effective approach [32]. Another study in Indonesia initiated a FA Guideline application on smartphones to improve access to FA knowledge among children aged 11–14 years [33].

Additionally, in the group with prior training experience, over 60% of these participants received training over 1 year or could not recall when. This raised concerns about the retention of FA knowledge and skills among the students. The previous study on Malay adolescents found that the students with previous training experience provided the wrong answers and implemented the incorrect answers or wrong management [22]. These mistakes might be due to the retention of knowledge among students. Especially, this decline in practice was expected to be especially severe, as skills were more easily lost with time when not practiced. Therefore, learners were required to renew their CPR or BLS license every year [3].

However, the students with better knowledge and attitude scores at baseline did not show a significant association with the improvement in the FA skills after receiving FA training. The literature on the association between knowledge, attitude, and skills is varied. Several studies showed that learners with better knowledge will be more likely to gain better practice skills [11]. However, Wafik et al. found a lack of correlation between school students’ scores of knowledge and situational practice at any of the three intervention phases [3]. This was possible because the student with good knowledge might not ensure that they would have a good ability to perform FA. Particularly in our study, most students surveyed showed low scores of knowledge, and the significant difference in FA skills between groups was unclearly specified.

Our study had several limitations. First, our study did not include a control group due to the influence of COVID-19-related social isolation measures, which prevented us from conducting the reassessment phases in the control group. As a result, the effects of the training program might not be captured comprehensively and might be influenced by residual confounding. Due to the limitation of the single-group pre-post design, the causality of FA training and the improvement of FA skills should be interpreted cautiously. Future studies with a quasi-experimental design would be beneficial in drawing more robust effects of FA training on FA skills among students. However, the study still provided valuable evidence in evaluating the effect of first-aid training programs in Vietnamese high schools. Secondly, the measurement of the effects of the intervention on the occurrence and frequency of injuries was limited. Thirdly, we were not able to observe the long-term effect of the intervention due to the COVID-19 pandemic lockdown. Therefore, further studies might need to address these issues to provide more accurate impact of training on the retention of FA knowledge in high school students. Additionally, there is no standard checklist of FA practice among school children in low-middle-income countries. We developed the checklist based on international and national guidelines and advice from experts in resuscitation, with adaptation to the Vietnamese context based on our literature review. Due to a lack of a gold standard in evaluating FA skills in low-middle-income countries, comprehensive tests on validation and reliability were not implemented. Further studies focusing more on these aspects would be essential for a more valid evaluation.

## Conclusions

In conclusion, this pre-post-intervention study showed a relatively low level of FA knowledge, attitude, and practice among high school students in Hue City, Vietnam. The short course training on basic FA techniques showed its feasibility in improving the FA skills among the participants, especially chest compression and ventilation skills. Our FA training program can provide a valuable reference for health policymakers, FA trainers, and health educators to enhance their knowledge, attitude, and skills in school settings. To scale up the program’s impact, we recommend integrating FA knowledge into online platforms that the majority of children are using. For areas with limited internet access, the handbook of FA practice available in the school library could be a useful alternative for students. Regarding FA attitude and skills, the targeted training for high school teachers is important to integrate knowledge into the curriculum and ensure reinforced training for students.

## Supporting information

S1 Table The correlation matrix of the factors after the oblique rotation. (DOCX)

S2 Table The score of knowledge, attitude, and skills of participants in baseline phase. (DOCX)

S3 Table The skill score between pre- and post-intervention. (DOCX)

S4 Table Change of skill score after intervention by gender (n = 106). (DOCX)

S5 Table The scoring table of first aid knowledge. (DOCX)

S6 Table The item list of the first aid attitude. (DOCX)

S7 Table The checklist of the first aid skill. (DOCX)

S1 Fig The parallel analysis of efficacy scale. (TIF)

S2 Fig The score of first aid skill between pre- and post-intervention phase among the male and female participants (n = 106). (TIF)

S1 Appendix FA training lesson plan. (DOCX)

S2 Appendix STROBE Checklist for the cross-sectional study. (DOCX)

S3 Appendix Diagnostic test. (DOCX)
