# Supplementary material for: To save or not to save: Knowledge, attitude, skills and effects of an experimental intervention on advancing first aid skills in high school students in Hue City, Vietnam
Source: PLoS One. 2025 Apr 29;20(4):e0322505. doi: 10.1371/journal.pone.0322505 (PMC12040149; doi:10.1371/journal.pone.0322505)
Supplement: S1 Table — (DOCX) [file pone.0322505.s001.docx]

**S1 Table. The correlation matrix of the factors after the oblique rotation.**

| **Factors** | **Factor1** | **Factor2** | **Factor3** |
| --- | --- | --- | --- |
| Factor 1 | 0.749 | 0.68 | 0.744 |
| Factor 2 | -0.66 | 0.635 | 0.425 |
| Factor 3 | 0.051 | 0.367 | -0.515 |

Barthell test: p-value < 0.001

KMO = 0.72

Cronbach alpha = 0.73 (add Cronbach for each factor)
