# Supplementary material for: To save or not to save: Knowledge, attitude, skills and effects of an experimental intervention on advancing first aid skills in high school students in Hue City, Vietnam
Source: PLoS One. 2025 Apr 29;20(4):e0322505. doi: 10.1371/journal.pone.0322505 (PMC12040149; doi:10.1371/journal.pone.0322505)
Supplement: S2 Table — (DOCX) [file pone.0322505.s002.docx]

**S2 Table.** The range score of knowledge, attitude, and skills of participants in baseline phase

|  | **Possible range scale (unstandardized)** | **Min-max (standardized on the scale from 0 to 100 points)** |
| --- | --- | --- |
| **Overall knowledge** | 0-34.5 | 2.9-71.7 |
| Primary assessment and call ambulance | 0-9 | 0-100 |
| CPR | 0-10 | 0-90 |
| Bleeding control | 0-4.5 | 0-100 |
| Joint injury management | 0-2.5 | 0-100 |
| Fracture mobilization | 0-4 | 0-100 |
| Burn management | 0-4.5 | 0-88.9 |
| **Attitude** | 7-28 | 0-85.4 |
| **Overall skill score** | 0-50 | 12-84 |
| Primary assessment | 0-20 | 0-90 |
| Chest compression | 0-10 | 0-100 |
| Ventilation circulation | 0-12 | 0-100 |
| Bleeding control | 0-8 | 0-100 |
