# Supplementary material for: To save or not to save: Knowledge, attitude, skills and effects of an experimental intervention on advancing first aid skills in high school students in Hue City, Vietnam
Source: PLoS One. 2025 Apr 29;20(4):e0322505. doi: 10.1371/journal.pone.0322505 (PMC12040149; doi:10.1371/journal.pone.0322505)
Supplement: S3 Table — (DOCX) [file pone.0322505.s003.docx]

**S3 Table.** The skill score between pre- and post- intervention.

| **Data characteristics** | | **Total (n=106)** | | | **Male (n = 40)** | | | **Female (n= 66)** | | |
| --- | --- | --- | --- | --- | --- | --- | --- | --- | --- | --- |
|  |  | **Baseline** | **Intervention** | **Mean difference (SE) ^a^** | **Baseline** | **Intervention** | **Mean difference (SE) ^a^** | **Baseline** | **Intervention** | **Mean difference (SE) ^a^** |
| Overall skill score | Mean (SD) | 40.1  (11.4) | 77.1  (11.3) | 37.0  (1.6)^***^ | 40.5  (12.4) | 77.3  (10.5) | 36.8  (3.0)^***^ | 39.9  (10.9) | 77  (11.9) | 37.1  (1.8) ^***^ |
|  | Median (Q1, Q3) | 38  (32 – 48) | 78  (70 – 86) |  | 40  (33-48) | 76  (71-86) |  | 38  (32-50) | 80  (70-86) |  |
| Primary assessment | Mean (SD) | 50.3  (12.6) | 74.6  (12.6) | 24.3  (1.6) ^***^ | 49.8  (11.7) | 74.3  (11.9) | 24.5  (2.5) ^***^ | 50.6  (13.2) | 74.8  (13.1) | 24.2  (2.2) ^***^ |
|  | Median (Q1, Q3) | 50  (40 – 60) | 75  (65 – 85) |  | 47.5  (45-60) | 77.5  (65-85) |  | 50  (40-60) | 75  (65-85) |  |
| Chest compression | Mean (SD) | 16.9  (23.8) | 77.5  (17.9) | 60.6  (2.8) ^***^ | 23.5  (29.7) | 80.5  (17.2) | 57  (5.3) ^***^ | 12.9  (18.6) | 75.8  (18.2) | 62.9  (3.1) ^***^ |
|  | Median (Q1, Q3) | 0  (0-20) | 80  (70 – 90) |  | 20  (0-30) | 90  (70-90) |  | 0  (0-20) | 80  (70-90) |  |
| Ventilation | Mean (SD) | 23.5  (23.2) | 76.0  (19.9) | 52.5  (3.2) ^***^ | 23.5  (25.5) | 75.6  (21.5) | 52.1  (6.3) ^***^ | 23.5  (21.9) | 76.3  (19) | 52.8  (3.4) ^***^ |
|  | Median (Q1, Q3) | 16.7  (8.3 – 33.3) | 75  (66.7 – 91.7) |  | 16.7  (0-37.5) | 79.2  (66.7-91.7) |  | 16.7  (8.3-33.3) | 75  (66.7-91.7) |  |
| Stopping bleeding | Mean (SD) | 68.6  (25.4) | 84.4  (17.2) | 15.8  (2.7) ^***^ | 64.1  (27.5) | 83.1  (17.1) | 19.1  (4.9) ^***^ | 71.4  (23.8) | 85.2  (17.4) | 13.8  (3.1) ^***^ |
|  | Median (Q1, Q3) | 75  (50 – 87.5) | 87.5  (75 – 100) |  | 75  (50-75) | 87.5  (75-100) |  | 75  (62.5-87.5) | 87.5  (75-100) |  |

^a^ Wilcoxon tests were applied.

^***^ p < 0.001
