# Supplementary material for: To save or not to save: Knowledge, attitude, skills and effects of an experimental intervention on advancing first aid skills in high school students in Hue City, Vietnam
Source: PLoS One. 2025 Apr 29;20(4):e0322505. doi: 10.1371/journal.pone.0322505 (PMC12040149; doi:10.1371/journal.pone.0322505)
Supplement: S4 Table — (DOCX) [file pone.0322505.s004.docx]

**S4 Table.** Change of skill score after intervention by gender (n = 106)

|  | **Pre-intervention Mean (SD)** | **Post-intervention Mean (SD)** | **Mean Differences (post and pre-intervention)** | **p value ^a^** |
| --- | --- | --- | --- | --- |
| **Total** | | | | |
| Overall skill score | 46.4 (2.1) | 62.2 (2.2) | 15.8 (3.1) | <0.001 |
| Primary assessment | 50.5 (2) | 74.3 (2) | 23.8 (2.7) | <0.001 |
| Chest compression | 16.9 (2.3) | 67.9 (2.5) | 51.0 (3.3) | <0.001 |
| Ventilation | 28.2 (2.7) | 73.8 (2.1) | 45.6 (3.6) | <0.001 |
| Stopping bleeding | 68.6 (2.5) | 75.1 (2.7) | 6.5 (3.2) | 0.048 |
| **Female** | | | | |
| Overall skill score | 45.9 (2.6) | 62 (2.9) | 16.1 (3.5) | <0.001 |
| Primary assessment | 51 (2.7) | 74.6 (2.7) | 23.6 (3.6) | <0.001 |
| Chest compression | 12.9 (2.3) | 65.4 (3.2) | 52.5 (3.9) | <0.001 |
| Ventilation | 28.2 (3.2) | 74.1 (2.6) | 45.9 (3.9) | <0.001 |
| Stopping bleeding | 71.4 (2.9) | 76.4 (3.4) | 5.0 (3.8) | 0.2 |
| **Male** | | | | |
| Overall skill score | 47.1 (3.8) | 62.5 (3.3) | 15.4 (5.8) | 0.01 |
| Primary assessment | 49.6 (3.1) | 73.8 (3.1) | 24.2 (4.2) | <0.001 |
| Chest compression | 23.5 (4.7) | 72.1 (3.9) | 48.6 (6.0) | <0.001 |
| Ventilation | 28.3 (4.8) | 73.4 (3.7) | 45.2 (7.2) | <0.001 |
| Stopping bleeding | 64.1 (4.3) | 73 (4.3) | 8.9 (5.8) | 0.133 |

^a^ Wilcoxon tests were applied.
