# Supplementary material for: To save or not to save: Knowledge, attitude, skills and effects of an experimental intervention on advancing first aid skills in high school students in Hue City, Vietnam
Source: PLoS One. 2025 Apr 29;20(4):e0322505. doi: 10.1371/journal.pone.0322505 (PMC12040149; doi:10.1371/journal.pone.0322505)
Supplement: S5 Table — (DOCX) [file pone.0322505.s005.docx]

**S5 Table.** The scoring table of first aid knowledge

| **Questions** | **Score** |
| --- | --- |
| 1.The first step is needed to implement when encountering an accident. | 1 |
| 2. What is the sequence of practicing the principle of DRCAB when encountering an unconscious victim | 1 |
| 3. What is the best approach to check if the victim is unconscious? | 1 |
| 4. Which part of the body could you observe to evaluate whether the victim has stopped breathing? | 1 |
| 5. How could you check the victim's pulse? | 1 |
| 6. After the primary assessment is complete, which sequence should you prioritize in managing the victim? | 1 |
| 7. If calling an emergency is essential, what should you do? | 1 |
| 8. What is the phone number to call an ambulance? | 1 |
| 9. If the victim is unconscious but breathing, how would you put the victim in a recovery position? | 1 |
| 10. When is CPR needed for the victim? | 1 |
| 11. What is the best position for the victim when performing CPR? | 1 |
| 12. What is the rate of chest compressions per minute for an adult victim? | 1 |
| 13. How deep is effective when performing chest compressions in adults? | 1 |
| 14. Why should we wait for the chest to spring back (recoil) after each push? | 1 |
| 15. What position to place your hands in when performing chest compressions? | 1 |
| 16. For those not proficient in CPR, which of the following practice would be recommended? | 1 |
| 17. What should you do to clear the victim's airway? | 1 |
| 18. What should you notice before performing ventilation? | 1 |
| 19. What is the ratio of chest compressions and CPR for adults? | 1 |
| 20. If the victim suffered severe bleeding in the hands or feet, what should you do (multiple options)? | 1.5 |
| 21. Which steps of immediate first aid should we do for a person who suffers from heavy blood loss? | 1 |
| 22. Which following practice is part of nose bleeding first aid? | 1 |
| 23. If a deep wound was caused by rusty metal objects, what should be noted in addition to cleaning and dressing the wound? | 1 |
| 24. If your leg is sprained or strained, what should you do? | 0.5 |
| 25. If an ice pack is available, where should the ice pack be placed? | 1 |
| 26. What should you do with a patient who just experienced dislocated ankle? | 1 |
| 27. What is the most significant indicator that a victim has a broken arm when you first encounter that person? | 1 |
| 28. What is the purpose of the immobilization of broken bones? Please select the best answer. | 1 |
| 29. What should we do when suspecting a victim has broken a bone? | 1 |
| 30. Which following cases are suspected for a victim with spinal cord injury? | 1 |
| 31. When your forearm was burned by boiling water, How long should a burn be cooled under water? | 1 |
| 32. In your opinion, which of the following substances and solutions can be applied to the burn areas? ( many choices) | 0.5 |
| 33. If there are blisters in the burn area what should you do? | 1 |
| 34. Is it necessary to cover the burn victim's surface? | 1 |
| 35. Should you remove clothing and jewelry from the victim's burn area? | 1 |
