# Supplementary material for: To save or not to save: Knowledge, attitude, skills and effects of an experimental intervention on advancing first aid skills in high school students in Hue City, Vietnam
Source: PLoS One. 2025 Apr 29;20(4):e0322505. doi: 10.1371/journal.pone.0322505 (PMC12040149; doi:10.1371/journal.pone.0322505)
Supplement: S6 Table — (DOCX) [file pone.0322505.s006.docx]

**S6 Table.** The item list of the first aid attitude.

| **First aid attitude** | **Totally disagree (1 point)** | **Disagree (2 points)** | **Neutral (3 points)** | **Agree (4 points)** | **Totally agree (5 points)** |
| --- | --- | --- | --- | --- | --- |
| I confidently perform first aid for person who need help. |  |  |  |  |  |
| I confidently perform all first aid skills. |  |  |  |  |  |
| I confidently perform several first aid skills. |  |  |  |  |  |
| It is important to learn first aid. |  |  |  |  |  |
| It is neccesary to train students for first aid skills. |  |  |  |  |  |
| I am willing to perform first aid on strangers. |  |  |  |  |  |
| I will perform first aid when other people are present |  |  |  |  |  |
