# Supplementary material for: To save or not to save: Knowledge, attitude, skills and effects of an experimental intervention on advancing first aid skills in high school students in Hue City, Vietnam
Source: PLoS One. 2025 Apr 29;20(4):e0322505. doi: 10.1371/journal.pone.0322505 (PMC12040149; doi:10.1371/journal.pone.0322505)
Supplement: S7 Table — (DOCX) [file pone.0322505.s007.docx]

**S7 Table. The checklist of the first aid skill.**

| **Evaluated techniques** | **Excellent**  **( 2 points)** | **Good**  **(1 point)** | **Poor**  **(0 point)** | **Total score** |
| --- | --- | --- | --- | --- |
| **Round 1: primary assessment** | | | | |
| Assessing the danger of the accident situation |  |  |  |  |
| Evaluating the victim response |  |  |  |  |
| Calling support |  |  |  |  |
| Checking pulse |  |  |  |  |
| Checking the airway |  |  |  |  |
| Checking the respiratory |  |  |  |  |
| Dialling the ambulance number |  |  |  |  |
| Providing information on accident scenarios |  |  |  |  |
| Describing the victim condition |  |  |  |  |
| **Round 2: CPR** | | | | |
| **Chest compression** | | | | |
| Placing the hands on the right site. |  |  |  |  |
| Putting the victims in the right position. |  |  |  |  |
| Performing the adequate the rate of chest compression. |  |  |  |  |
| Compressing the adequate depth (5-6cm). |  |  |  |  |
| Waiting for chest recoil after each compression. |  |  |  |  |
| **Ventilation** | | | | |
| Opening the victim's airway, and tilt the head to one side to remove foreign bodies. |  |  |  |  |
| Performing head tilt and chin lift. |  |  |  |  |
| Taking a breath and placed our mouth on the victim’s mouth to blow. |  |  |  |  |
| Using one hand to squeeze both sides of the victim's nose. |  |  |  |  |
| Checking that victims’ chest rises. |  |  |  |  |
| Give 2 breaths at a rate of 8-10 times per minute. |  |  |  |  |
| **Round 3: Stopping bleeding in hands/ arms** | | | | |
| Assessing the bleeding wound |  |  |  |  |
| Using gauze/clean cloth to cover the wound |  |  |  |  |
| Pressing the wound with tape or cloth |  |  |  |  |
| Making an arm sling and an elevation sling with triangular bandage |  |  |  |  |
