# Supplementary figures and images for: To save or not to save: Knowledge, attitude, skills and effects of an experimental intervention on advancing first aid skills in high school students in Hue City, Vietnam
Source: PLoS One. 2025 Apr 29;20(4):e0322505. doi: 10.1371/journal.pone.0322505 (PMC12040149; doi:10.1371/journal.pone.0322505)

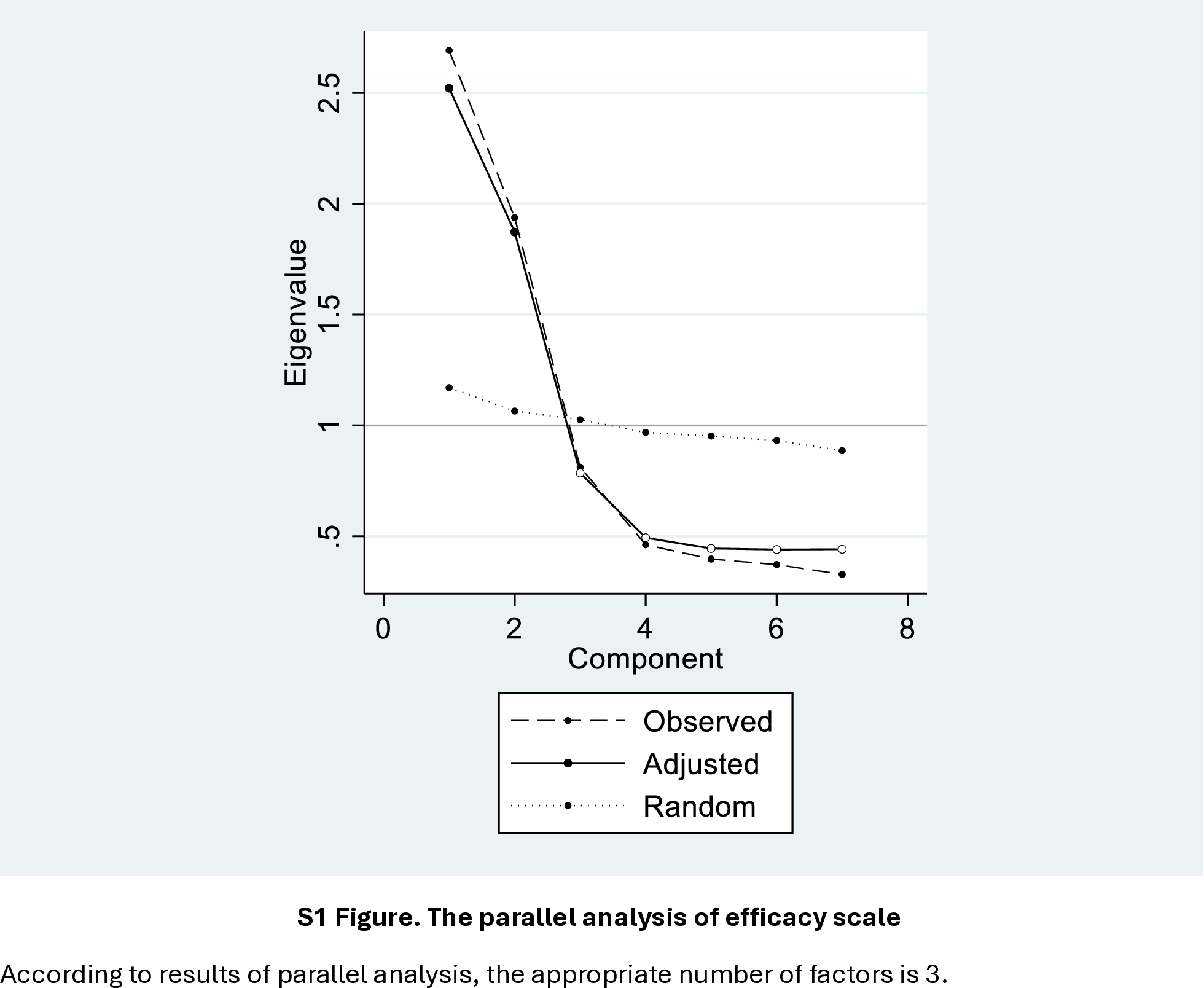

Supplement: S1 Fig — (TIF) [file pone.0322505.s008.tif]

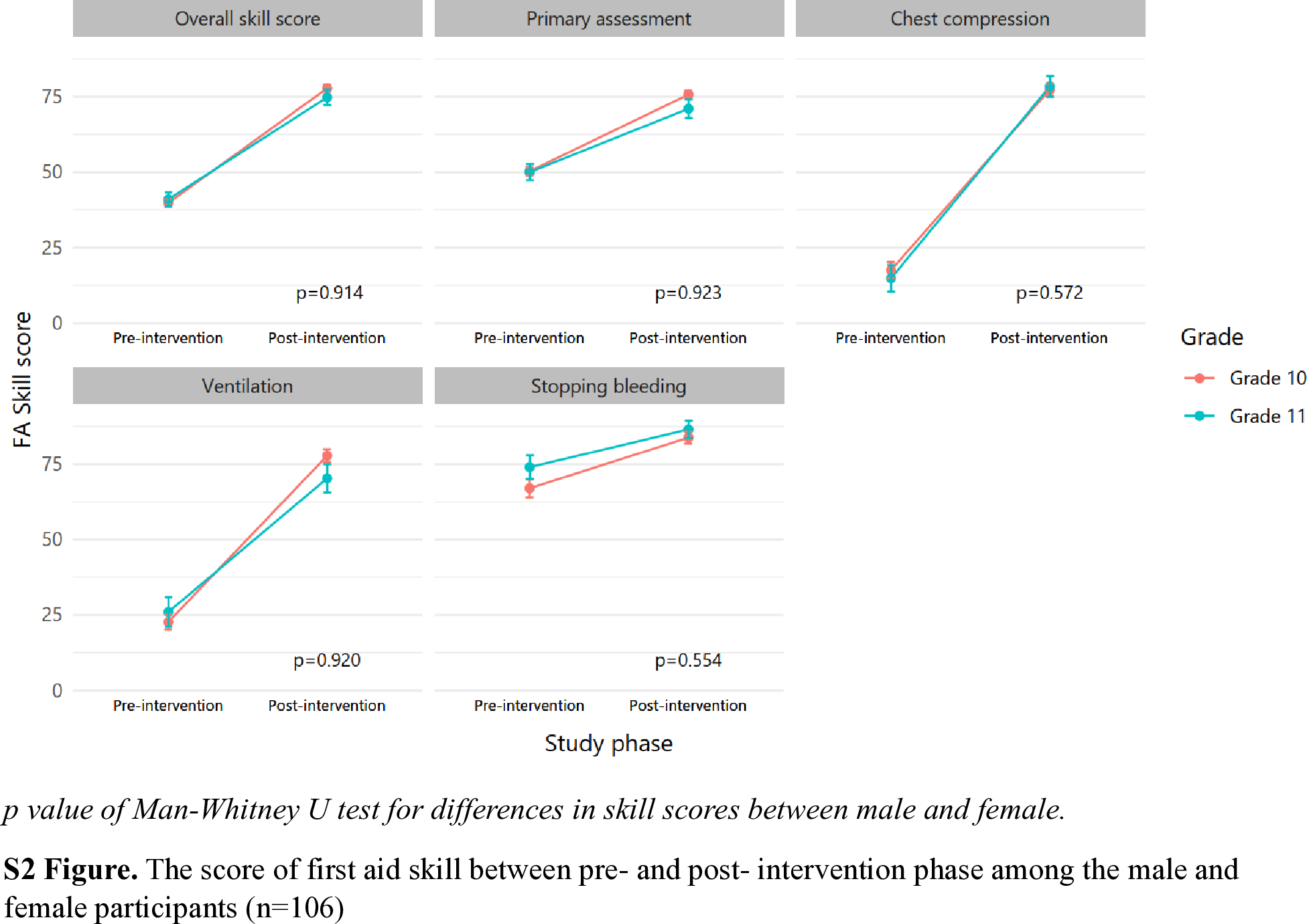

Supplement: S2 Fig — (TIF) [file pone.0322505.s009.tif]
