# Supplementary material for: To save or not to save: Knowledge, attitude, skills and effects of an experimental intervention on advancing first aid skills in high school students in Hue City, Vietnam
Source: PLoS One. 2025 Apr 29;20(4):e0322505. doi: 10.1371/journal.pone.0322505 (PMC12040149; doi:10.1371/journal.pone.0322505)
Supplement: S1 Appendix — (DOCX) [file pone.0322505.s010.docx]

**S1 APPENDIX. INSTRUCTION ON BASIC FIRST AID SKILLS FOR HIGH SCHOOL STUDENTS**

**Learning Objectives:** At the end of the lesson, students will be able to:

1. Understand the principles of first aid.

2. Understand the basic first aid steps in some situations where first aid equipment is not available.

**1. Basic information**

Location: Classroom

Number of participants: 25 students – divided into 4 groups (6-7 students/group)

Number of instructors: 1

Number of teaching assistants (TAs): 5

Number of sessions: 1 session – duration of session: 3 hours

**2. Training script for instructor**

**2.1. Warm-up: What is an emergency situation? (15 minutes)**

- Introduce the training program and team members.

- Play a warm-up game: Students are divided into 4 groups. Each group receives 6 images with the following topics:

+ Breaking time in the school yard

+ Going camping

+ Making instant noodles

+ Eating fruit with family

+ Ambulance carrying patients

+ Playing soccer next to the lake

- Groups arrange these images into a Venn diagram (maybe in the safe/ emergency/ intersection area)

What do you think makes these situations dangerous?

**2.2. What are the changes that will turn a fun activity into a serious one?**

- Are there any cases where safe situations can become dangerous situations? In what cases? (The instructors write down emergency phrases/words – save this list)

- From the above example, can we define an emergency situation?

- Definition of an emergency situation: a person is in an emergency situation when a situation poses a health risk, property damage and it cannot be resolved with normal resources”.

=> The instructor emphasizes to students: an accident/emergency situation can happen at any time, a fun moment can completely turn into an unexpected accident.

**2.3. Understanding risk: What makes a situation dangerous or unsafe?**

- What makes the above situations dangerous?

(List the reasons for dangerous situations)

- What can we do to avoid this dangerous situation?

(List measures to make the situation safe)

**2.4. Why and how do we prepare for an emergency?**

- With the following 4 emergency situations, how would you handle it?

(Each group will be shown 1 of the 4 situations by the TAs: encountering a heart attack, cardiac arrest, burns, bleeding and broken arm)

- Why do you do that? The group members discuss for 5 minutes and write down the group's answers?

(The TAs give each group a piece of A0 paper, ask a group representative to write down the discussion results.)

- Why is this situation urgent? How dangerous is it?

(After 5 minutes of discussion, all group discussions will be recorded.)

- In order, open each group's situation video for the whole class to watch and invite a group representative to present.

(The instructor will post the group discussion on the board)

- Do the remaining 3 groups have any additional comments to add to that group's answer?

(The TAs records the additional answer).

- The instructor gives a solution to the situation (answer).

- Are there any similarities or differences between the group's ideas and the answer? Is there anything that surprises you?

(Rotate each group)

**2.5. Game – Review what you have learned**

Groups will participate in the game with Plickers which includes 12 questions to review knowledge.

(Each student will receive a hard paper to show the answer)

The group with the highest points will receive a gift.

**2.6. Practice of emergency management (60 minutes)**

- 4 training skills: assessing consciousness - vital signs of the victim, CPR, first aid for broken bones, first aid for bleeding.

- Demonstration: Students watch videos, and the instructor demonstrates each step

(each group will receive a mankini model to practice)

+ Practice: Students practice one by one on the model and are supported by TAs to correct errors. (during the practice, instruction videos are shown on the screen)

+ Evaluation and experience: After completing 4 skills, each student will be evaluated on all the skills learned by TAs.

(The student with the highest score in each group will receive a gift)

**2.7. Summary of the lesson with key messages (15 minutes)**

- Risk assessment and preparation for first aid

- Principles of first aid.

- Introduce the first aid handbook and other sources of information to update first aid knowledge.

**2.8. Learner’s feedback (10 minutes)**
