# Supplementary material for: To save or not to save: Knowledge, attitude, skills and effects of an experimental intervention on advancing first aid skills in high school students in Hue City, Vietnam
Source: PLoS One. 2025 Apr 29;20(4):e0322505. doi: 10.1371/journal.pone.0322505 (PMC12040149; doi:10.1371/journal.pone.0322505)
Supplement: S3 Appendix — (DOCX) [file pone.0322505.s012.docx]

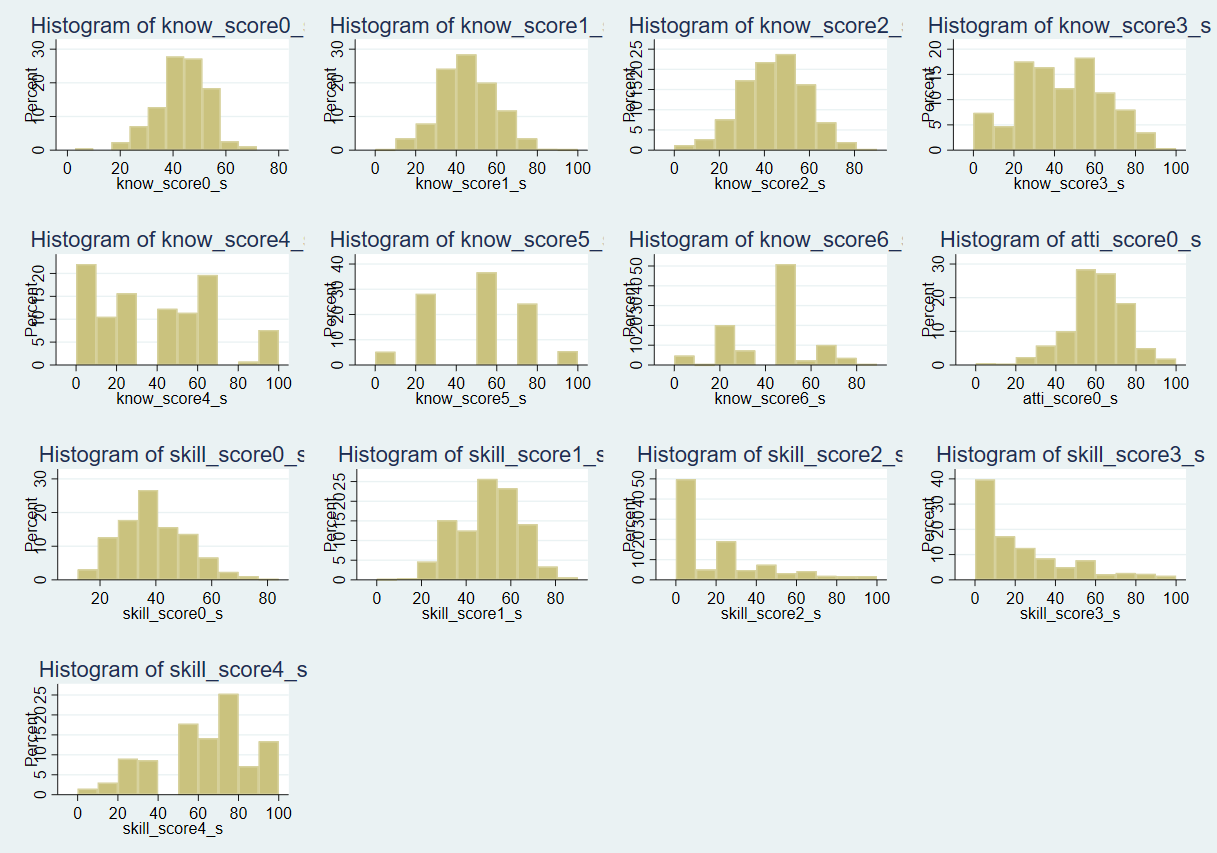


S1 Figure. The histograms of FA knowledge, attitude and skill scores at the baseline

Abbreviations: know_score0_s: Overall knowledge score; know_score1_s: primary assessment and call ambulance; know_score2_s: CPR; know_score3_s: Stop heavy bleeding and nose bleeding; know_score4_s: Joint disclosing; know_score5_s: Fracture mobilization; know_score6_s: Burn; atti_score0_s: Attitude towards first aid; skill_score0_s: Overall skill score; skill_score1_s: primary assessment and call ambulance; skill_score2_s: CPR; skill_score3_s: Stop heavy bleeding and nose bleeding; skill_score4_s: Joint disclosing; skill_score5_s: Fracture mobilization; skill_score6_s: Burn.


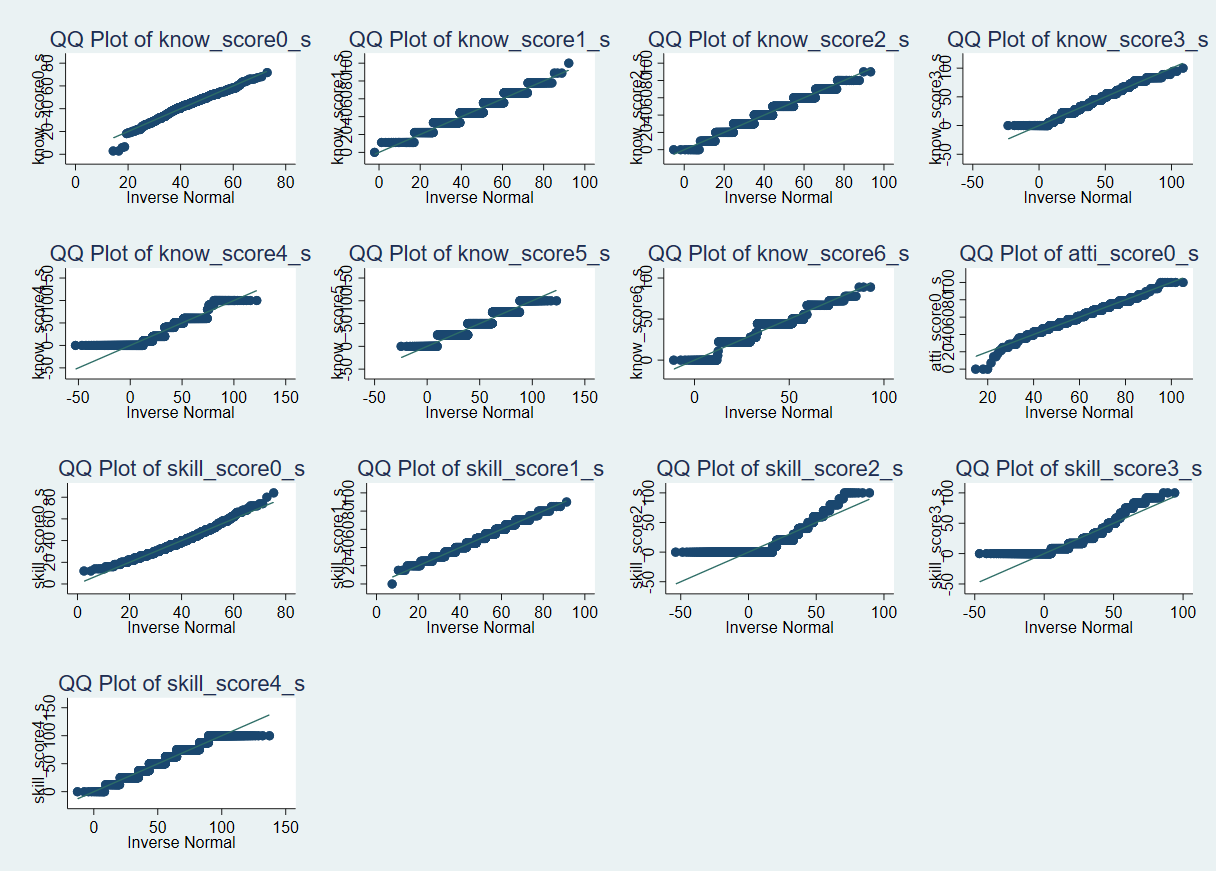


S2 Figure. The QQ Plot of FA knowledge, attitude and skill scores at the baseline

Abbreviations: know_score0_s: Overall knowledge score; know_score1_s: primary assessment and call ambulance; know_score2_s: CPR; know_score3_s: Stop heavy bleeding and nose bleeding; know_score4_s: Joint disclosing; know_score5_s: Fracture mobilization; know_score6_s: Burn; atti_score0_s: Attitude towards first aid; skill_score0_s: Overall skill score; skill_score1_s: primary assessment and call ambulance; skill_score2_s: CPR; skill_score3_s: Stop heavy bleeding and nose bleeding; skill_score4_s: Joint disclosing; skill_score5_s: Fracture mobilization; skill_score6_s: Burn.

According to the S1 and S2 Figures in Appendix 3, we defined variables with non-normal distribution including join injury management (know_score4_s), burn management (know_score6_s), chest compression (skill_score2_s), ventilation circulation (skill_score3_s), bleeding control (skill_score4_s). To test the differences in these variables between males and females, we employed the Mann-Whitney-Wilcoxon test.

**Testing Normality assumption of dependent variables**

|  |  |
| --- | --- |
| 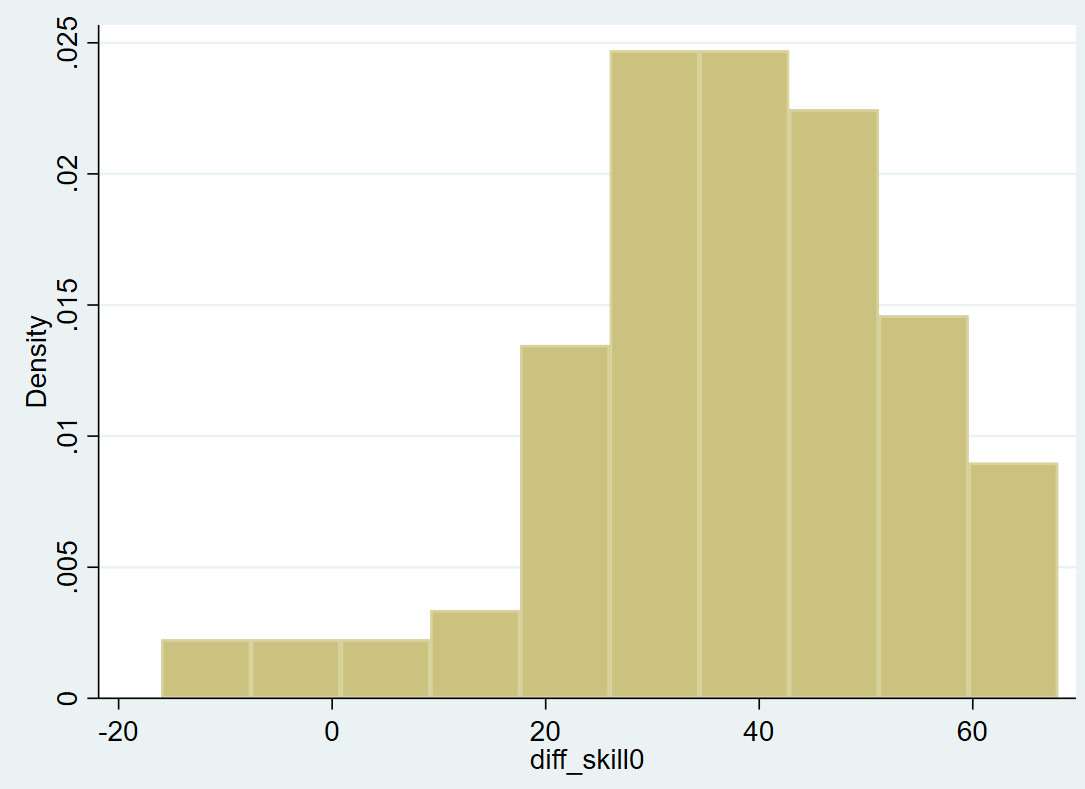 | 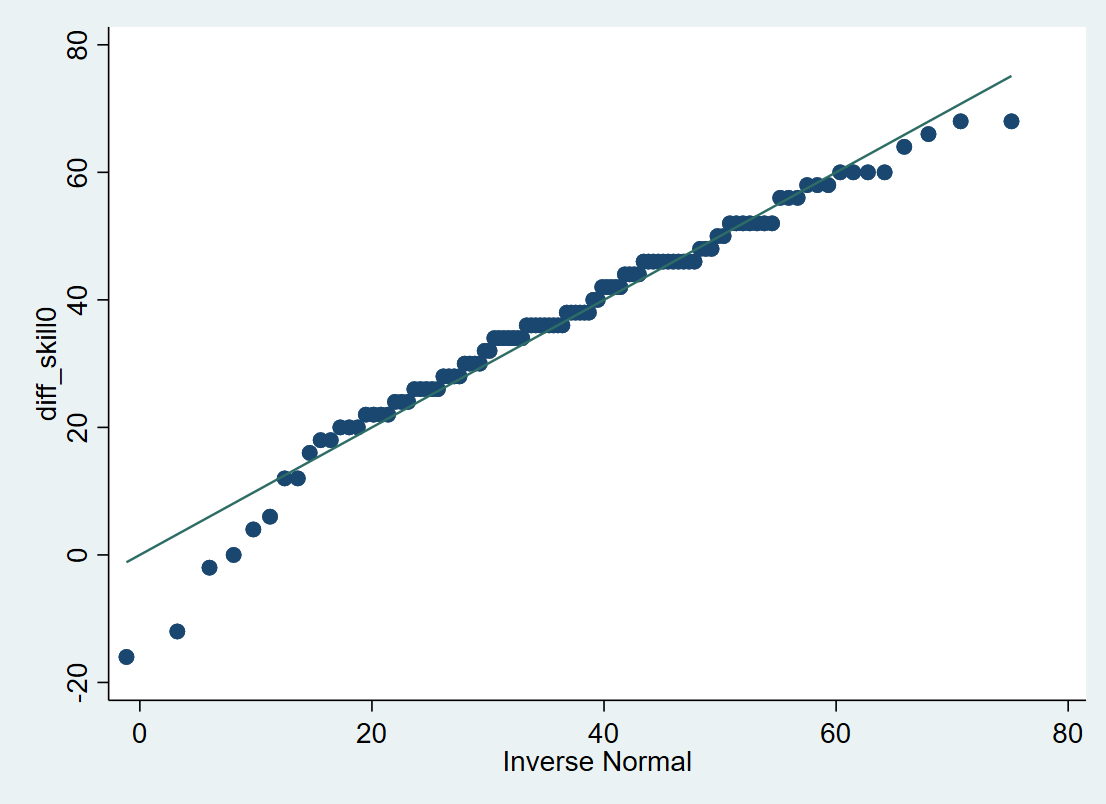 |
| Histogram of difference in skills score | QQ Plot of differences in skills score |
| 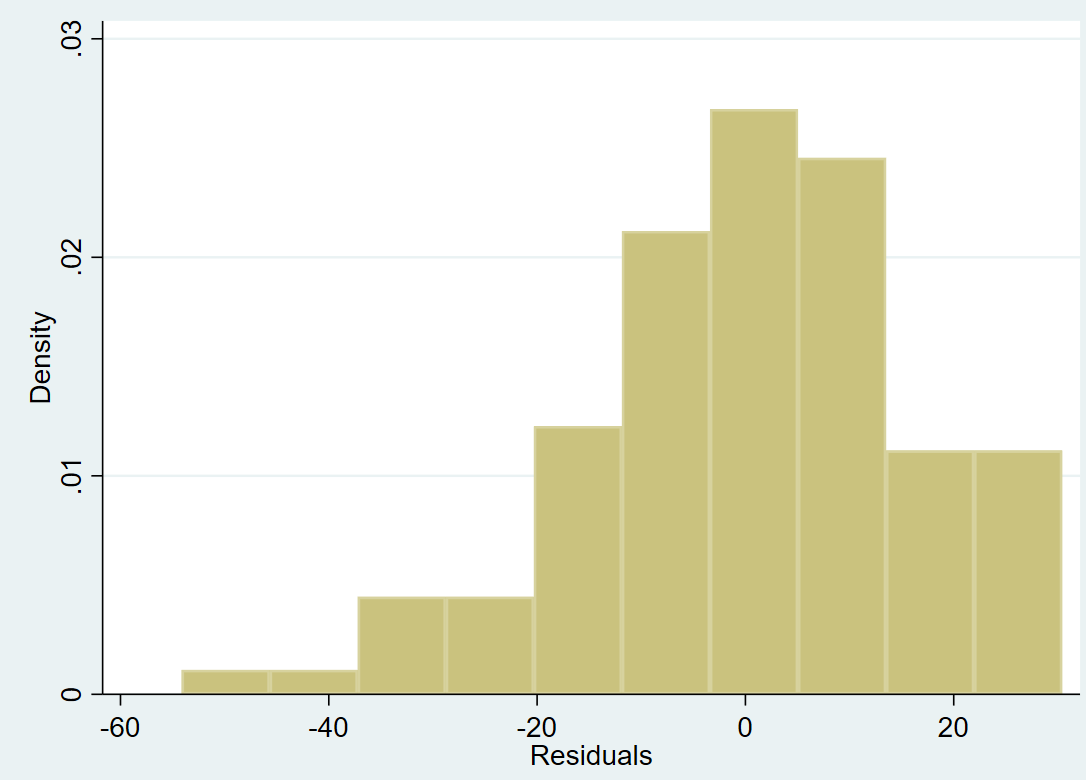 | 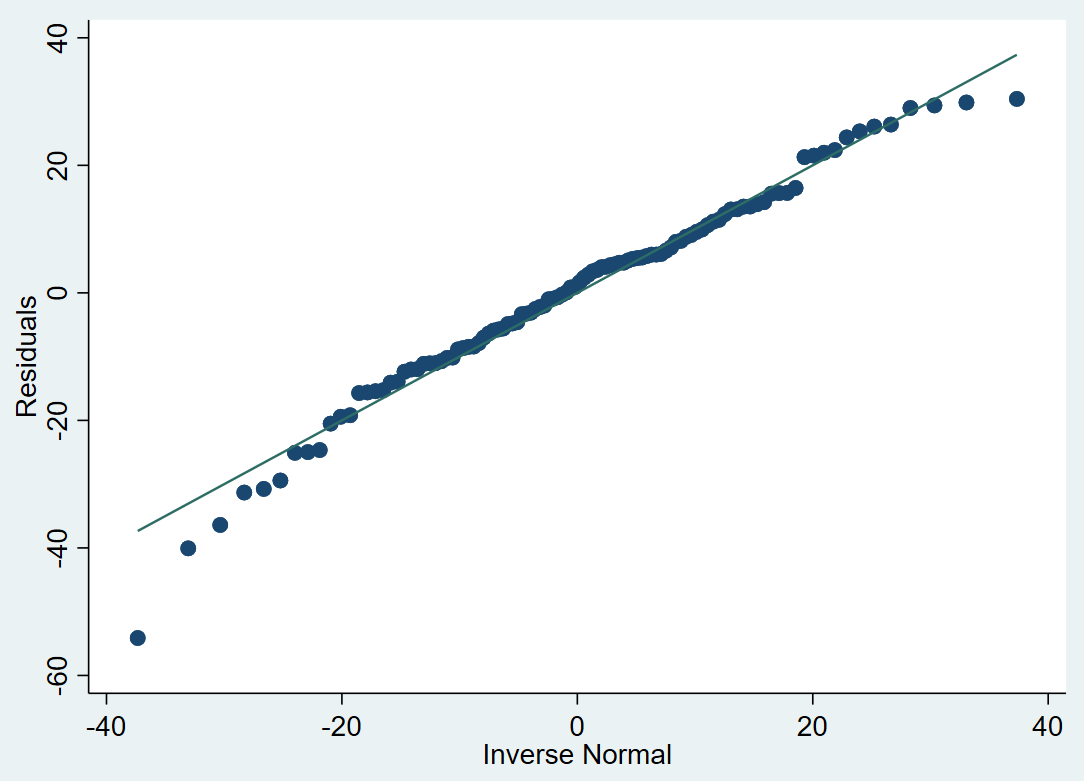 |
| Histogram of residuals for difference in skill scores | QQ plot of residuals for differences in skill scores |

S3 Figure. Histograms and QQ Plots of FA skill scores

Based on S3 Figure, a dependent variable (difference in FA skills score) was approximate to the normal distribution

**Testing assumption of homogeneity**


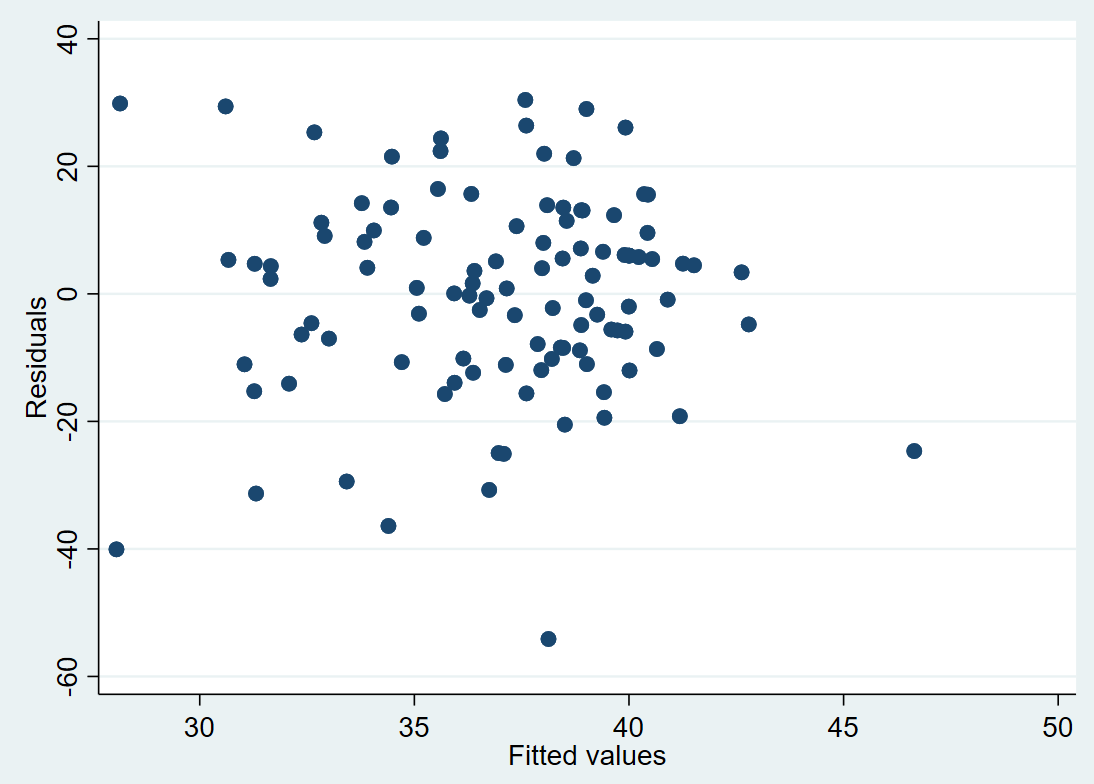


S4 Figure. Scatter plot of residuals versus fitted values

**S1 Table.** Testing heteroskedasticity of residuals using Cameron & Trivedi's decomposition of IM-test

| **Source** | **Chi2** | **df** | **P** |
| --- | --- | --- | --- |
| Heteroskedasticity | 30.62 | 23 | 0.132 |
| Skewness | 4.8 | 6 | 0.5696 |
| Kurtosis | 0.72 | 1 | 0.3951 |
| Total | 36.14 | 30 | 0.2035 |

The p value of the homogeneity test (Cameron & Trivedi's decomposition of IM-test) was 0.13 (S1 Table).

Based on the results of S1 Table and S4 Figure, there was no evidence to conclude that there was heterogeneity in our data.

**Testing assumptions of multicollinearity**

S2 Table. Variance Inflation Factor (VIF) of variables in regression models

| **Variables** | **VIF** |
| --- | --- |
| School names | 1.19 |
| Gender | 1.07 |
| Grade | 1.13 |
| Prior FA training | 1.11 |
| FA Attitude | 1.08 |
| FA Knowledge | 1.03 |

The result of S2 Table indicates very low multicollinearity among the predictors in the regression model since all individuals VIF values were around 1.

**Test assumptions of linearity**

| **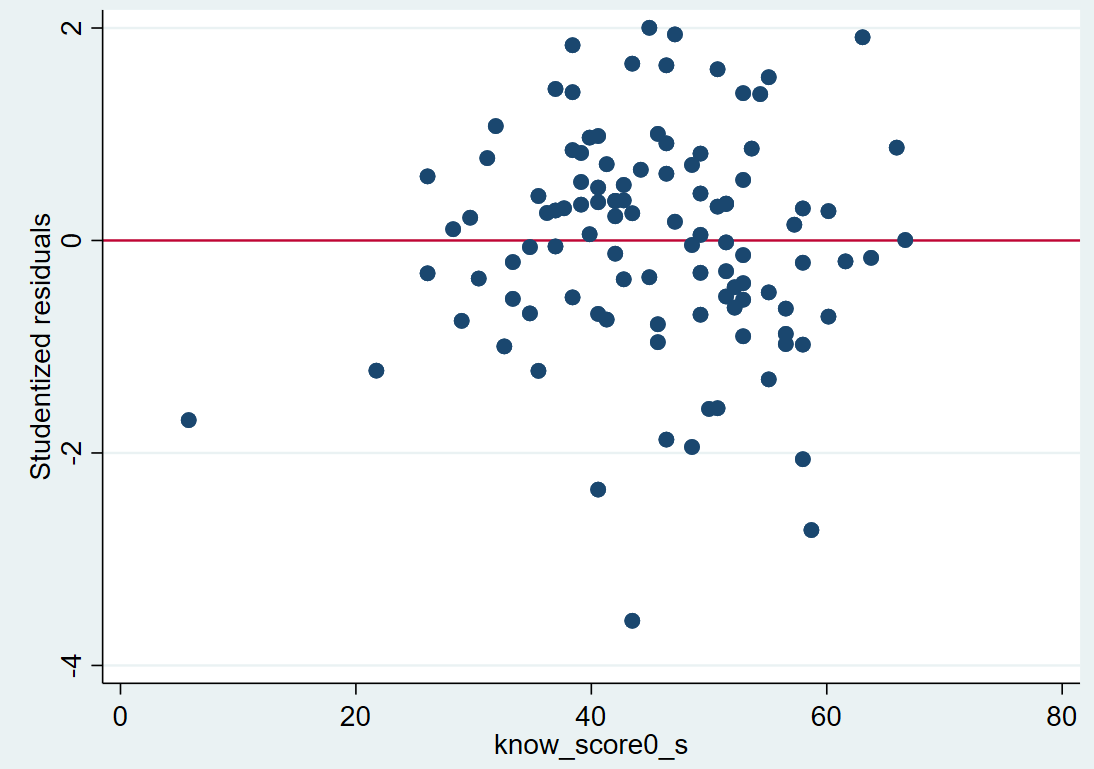** | **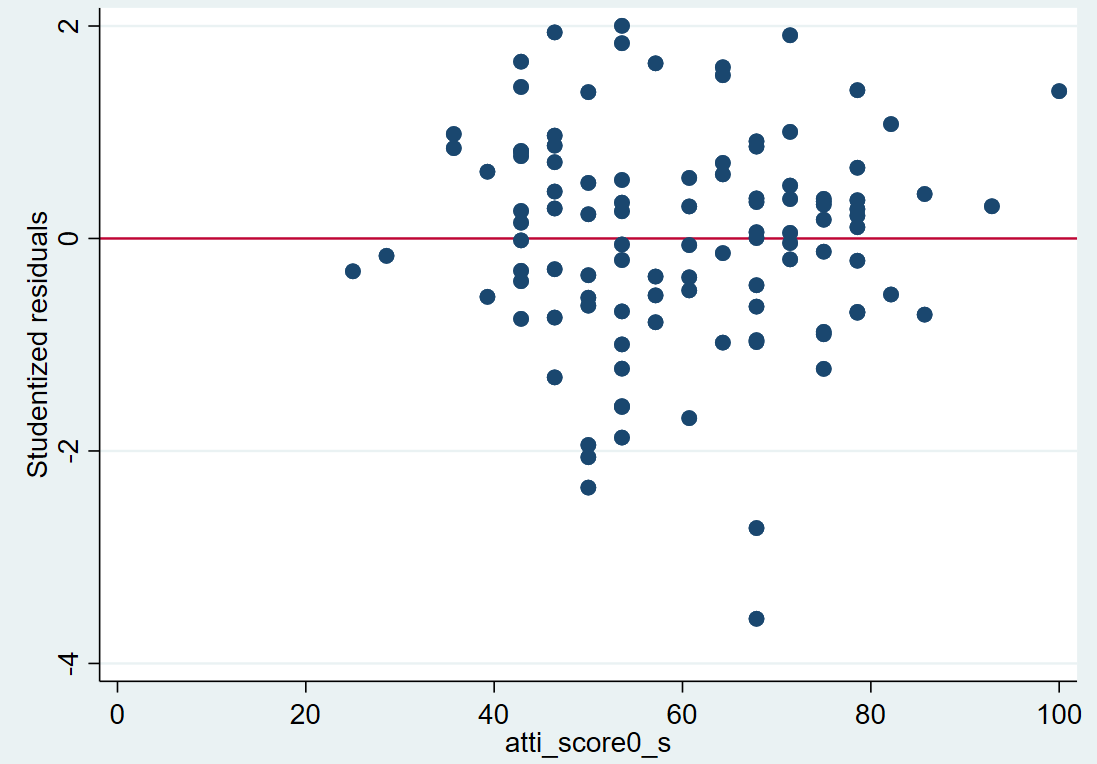** |
| --- | --- |

Abbreviations: know_score0_s: FA knowledge at baseline, atti_score0_s: FA attitude at baseline.

We carried out tests for the linearity with the continuous covariates in our regression model including FA knowledge at baseline, atti_score0_s: FA attitude at baseline. We plotted the studentized residuals against FA knowledge (know_score0_s) as well as FA attitude (atti_score0_s) at baseline to test the assumption. The result indicates that the scatter points are randomly distributed around zero with no evidence of curvature. Therefore, the linearity assumption appears to hold.

Conclusions: According to the above results of assumption testing (normality, heteroskedasticity, multicollinearity), the use of a linear regression model in our analysis was appropriate.
